# Supplementary figures and images for: Strategies for the production of isotopically labelled Fab fragments of therapeutic antibodies in Komagataella phaffii (Pichia pastoris) and Escherichia coli for NMR studies
Source: PLoS One. 2023 Nov 29;18(11):e0294406. doi: 10.1371/journal.pone.0294406 (PMC10686436; doi:10.1371/journal.pone.0294406)

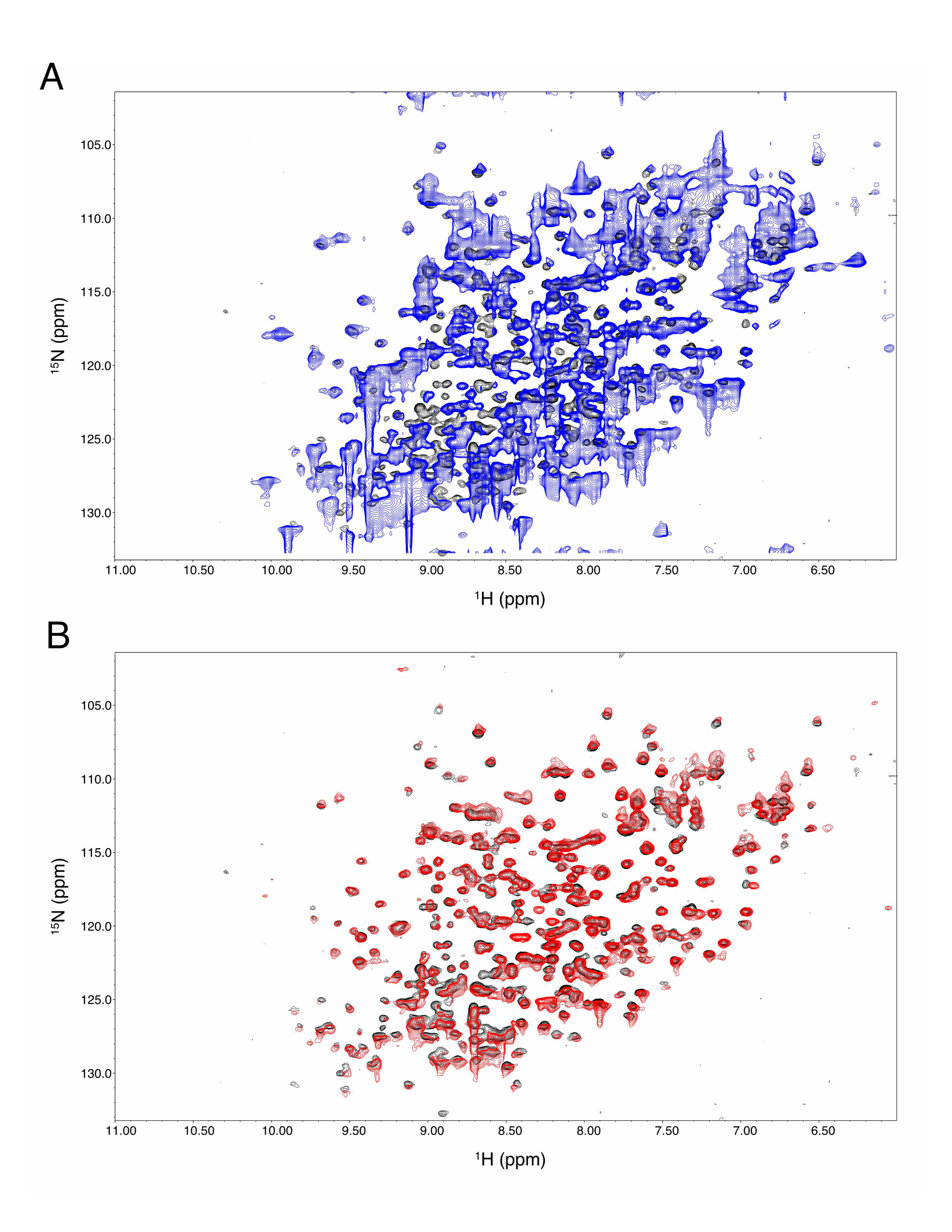

Supplement: S1 Fig — A) Overlay of 2D-1H-15N-SOFAST-HMQC of 15N-labelled isolate from yeast 15N-NIST-mAb-Fab isolated using cation exchange chromatography (blue) over NIST-mAb-Fab RM-8761 at natural abundance (black). The spectrum in blue shows high intensity peaks with distorted lineshape that are attributed to the light chain while peaks of lower intensities belong to the Fab. B) Overlay of yeast-produced 15N-NIST-mAb-Fab (red) and RM-8761 at natural abundance (black) showing the extra resonances arising from the tetrapeptides EAEA at the N-terminal end of the heavy and light chains. (JPG) [file pone.0294406.s001.jpg]

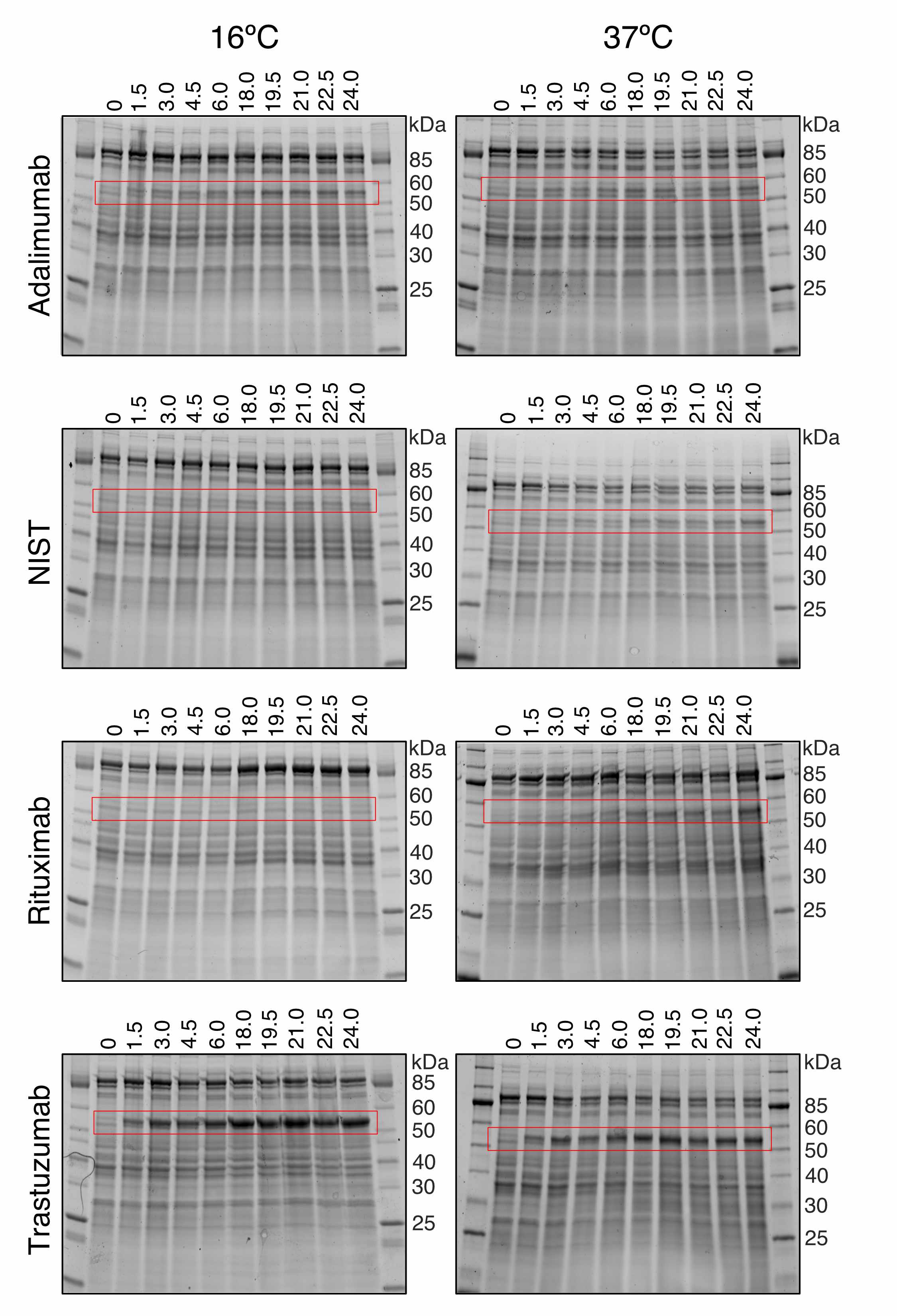

Supplement: S2 Fig — SDS-PAGE analysis of protein induction times (in hours indicated at the top of every gel) at two temperatures for all four Fab fragments. The target protein (histag-mAb-scFab c.a. 55 kDa) is boxed in red. (JPG) [file pone.0294406.s002.jpg]

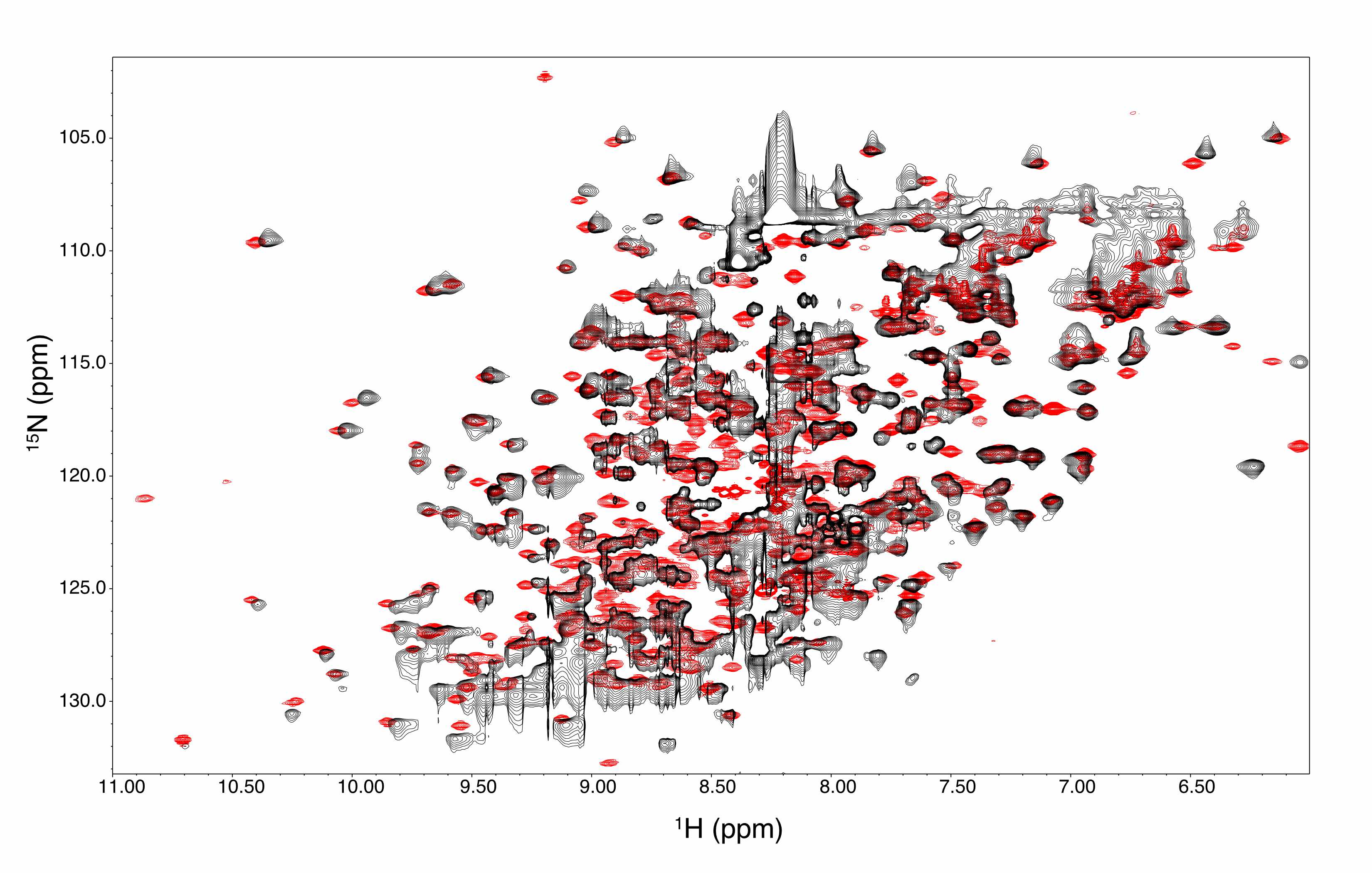

Supplement: S3 Fig — Overlay of 2D-1H-15N-SOFAST-HMQC of 15N-yeast-NISTmAb-Fab (red) and first very diluted sample of 15N-histag-NISTmAb-scFab prepared with IMAC on-column refolding from E. coli inclusion bodies (black). (JPG) [file pone.0294406.s003.jpg]

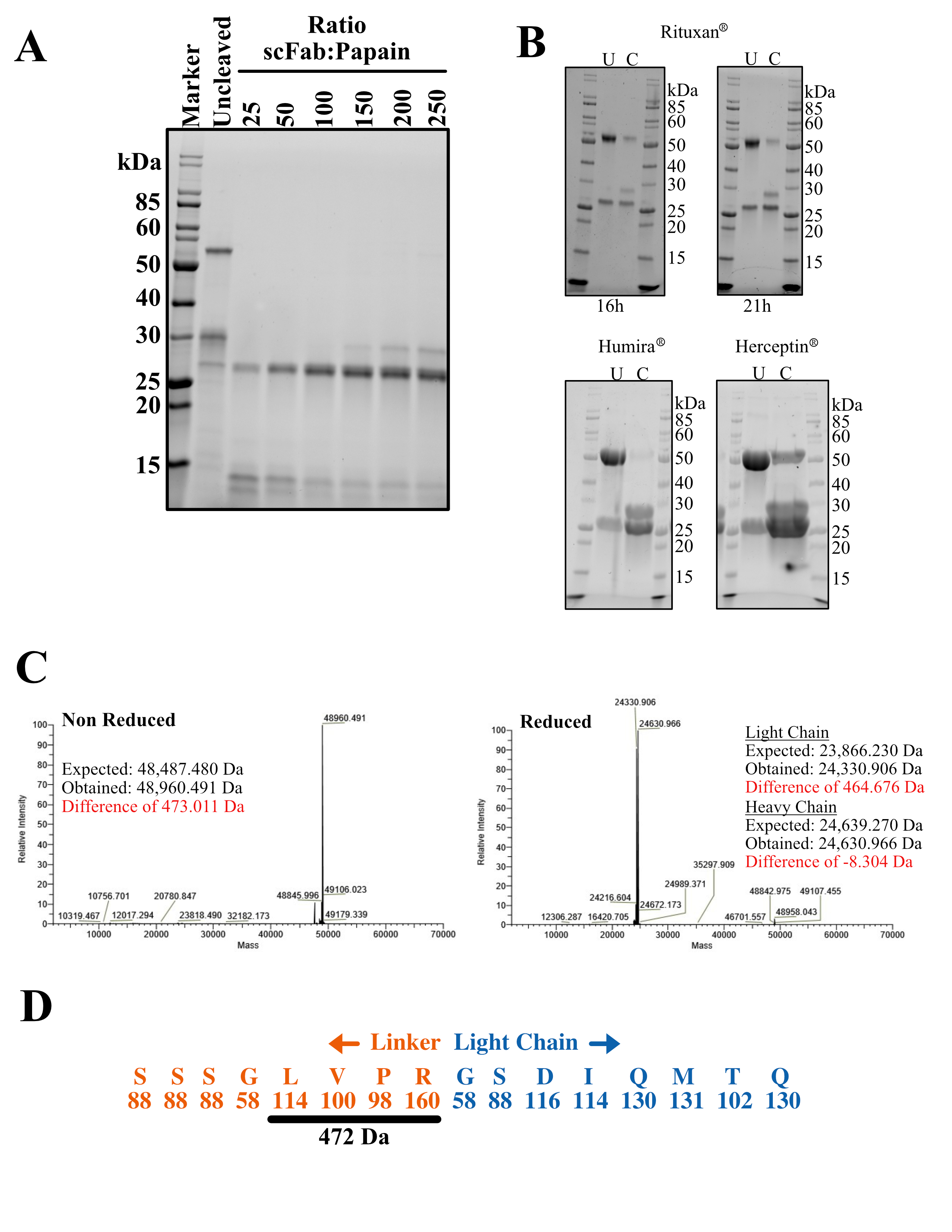

Supplement: S4 Fig — A) SDS-PAGE analysis of papain cleavage of histag-mAbs-scFabs. Ratios of scFab:Papain tested were 25:1;50:1, 100:1 and 250:1. B) SDS-PAGE analysis of papain cleavage of innovator therapeutic mAbs-Fabs. Letters U and C correspond to uncleaved and cleaved, respectively. C) Mass spectrometry analysis revealed an extra 472 mass units of the papain cleavage product on the non-reduced samples. Analysis of the reduced fragments shows that the extra residues are at the amino-terminal of the light chain. D) Amino acid sequence at the end of the linker and beginning of the light chain showing the mass of every residue underneath. (JPG) [file pone.0294406.s004.jpg]

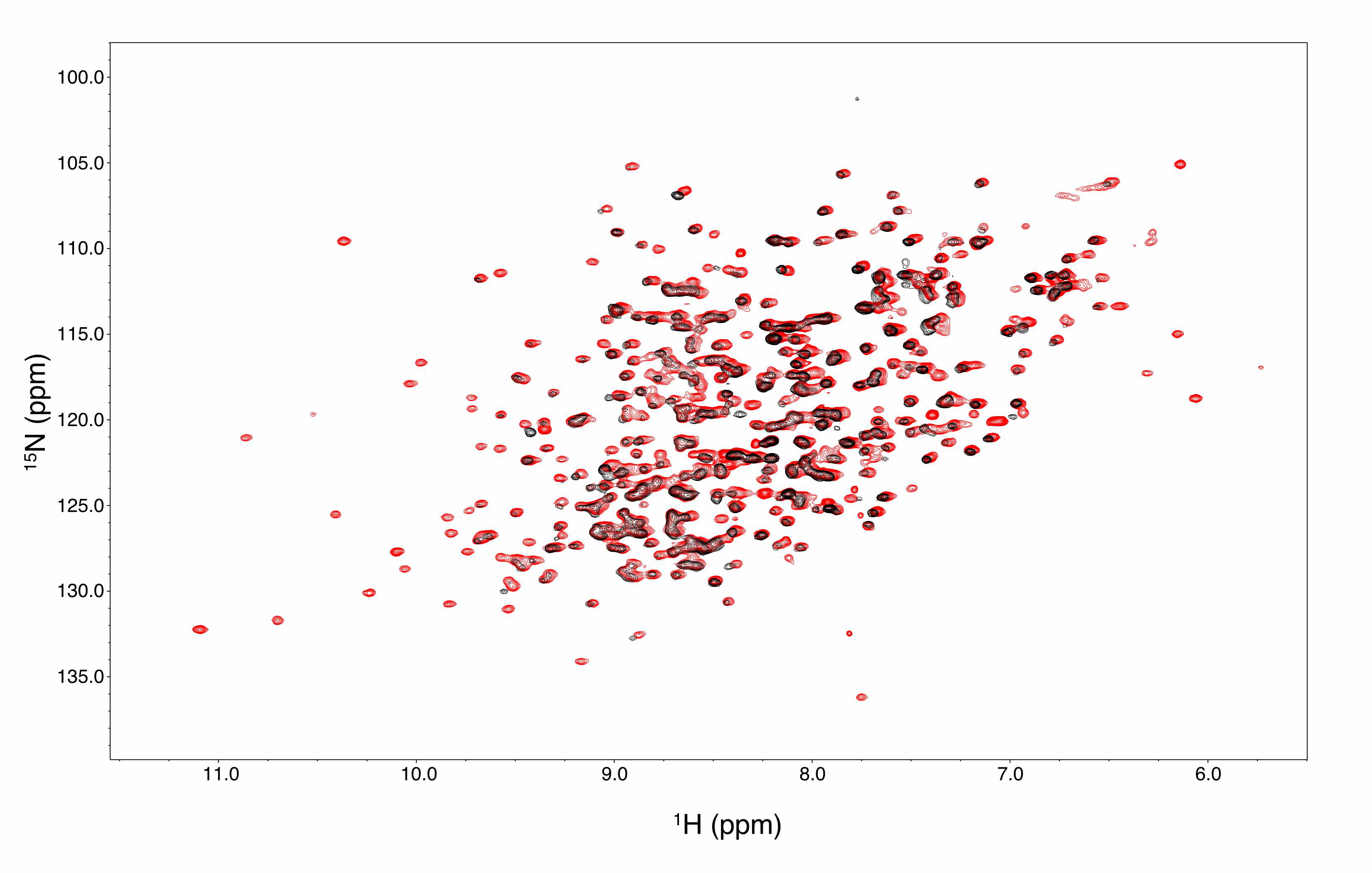

Supplement: S5 Fig — Overlay of 2D-1H-15N-HSQC of 15N-NISTmAb-Fab (tag and linker cleaved) (red) and 2D-1H-15N-SOFAST-HMQC of NIST-mAb-Fab RM-8761 at natural abundance (black). (JPG) [file pone.0294406.s005.jpg]

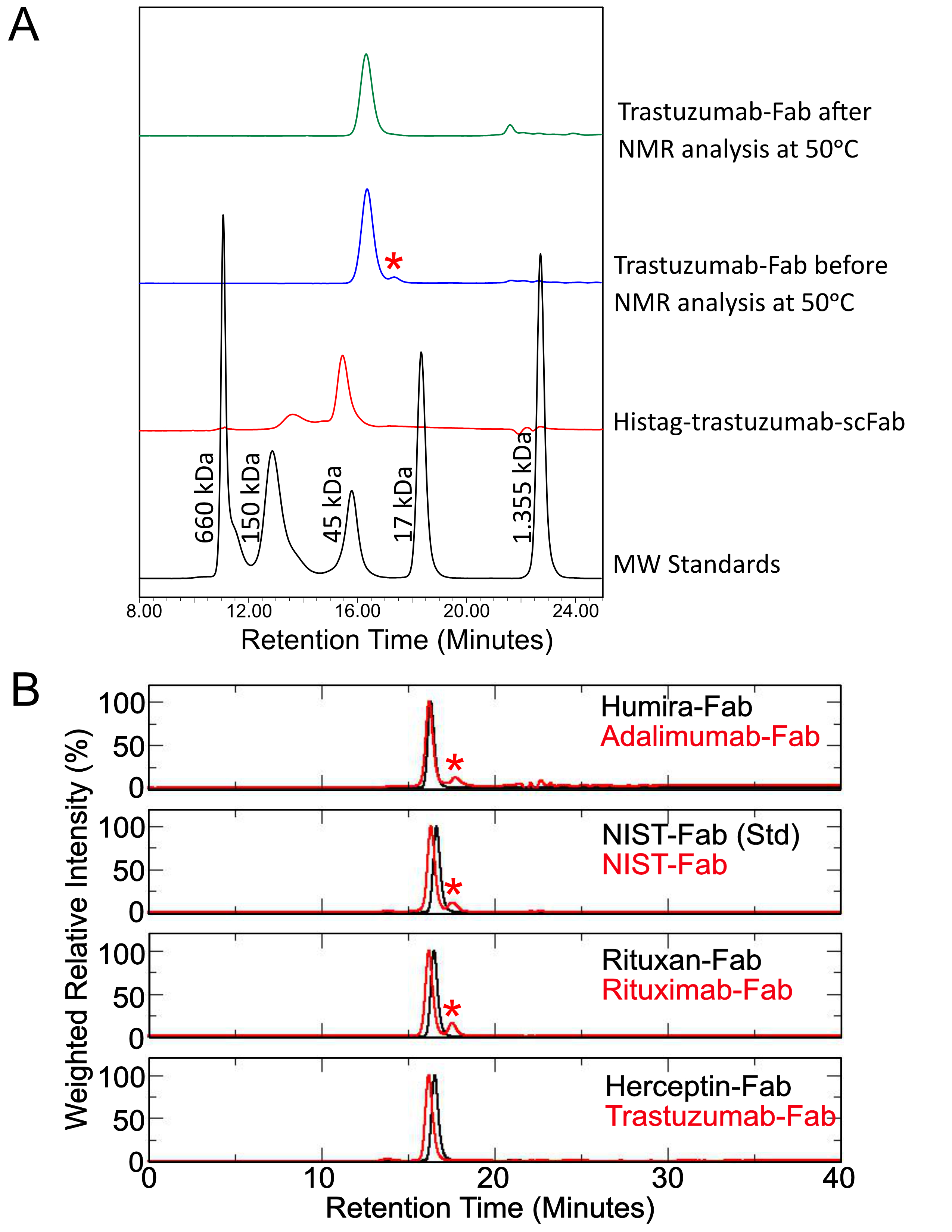

Supplement: S6 Fig — A) SEC analysis of E.coli prepared trastuzumab-Fab prior to cleavage of the histag and linker (histag-trastuzumab-scFab) and after cleavage (trastuzumab-Fab) freshly cleaved and after spending several hours in the NMR spectrometer at 50°C. Note the presence of an unknown impurity at ∼17.5min of lower molecular weight (denoted by a red star) that nearly disappeared after spending time at 50°C. B) Comparison of SEC analysis of E.coli prepared Fab and the Fab from the corresponding drug. In all preparations, but this preparation of trastuzumab, the impurity (red star) is present. (JPG) [file pone.0294406.s006.jpg]
