# Supplementary material for: Strategies for the production of isotopically labelled Fab fragments of therapeutic antibodies in Komagataella phaffii (Pichia pastoris) and Escherichia coli for NMR studies
Source: PLoS One. 2023 Nov 29;18(11):e0294406. doi: 10.1371/journal.pone.0294406 (PMC10686436; doi:10.1371/journal.pone.0294406)
Supplement: S1 Table — (DOCX) [file pone.0294406.s007.docx]

**Table S1**: Composition of the different vectors

|  | Host vector | Insert | Variation |
| --- | --- | --- | --- |
| Construct A | pPICZalphaA | NIST mAb Heavy Chain |  |
| Construct B | pPICZalphaA | NIST mAb Light Chain |  |
| Construct C | pPICZalphaA | NIST mAb Light Chain | pmeI site deleted |
| Construct D | pPICZalphaA | NIST mAb:  Heavy Chain and Light chain | bicistronic with 2 promotors |
| Construct E | pPICZalphaA | NIST mAb Fab:  Constant and variable domains of Heavy Chain and Light Chain | bicistronic with 2 promotors |
