# Supplementary material for: Strategies for the production of isotopically labelled Fab fragments of therapeutic antibodies in Komagataella phaffii (Pichia pastoris) and Escherichia coli for NMR studies
Source: PLoS One. 2023 Nov 29;18(11):e0294406. doi: 10.1371/journal.pone.0294406 (PMC10686436; doi:10.1371/journal.pone.0294406)
Supplement: S2 Table — (DOCX) [file pone.0294406.s008.docx]

**Table S2**: Sequences of the different primers

|  | Name for reference | Sequence |
| --- | --- | --- |
| Primer 1 | Pme Muta Fw | cccaa aactg acagt tcaaa cgctg tcttg gaacc |
| Primer 2 | Pme Muta rev | ggttc caaga cagcg tttga actgt cagtt ttggg |
| Primer 3 | Mut mAb Fab STOP Fw | gtctt gcgac aagac tcact aatgt ccacc atgtc ctgc |
| Primer 4 | Mut mAb Fab STOP Rev | gcagg acatg gtgga catta gtgag tcttg tcgca agac |
