# Supplementary material for: Strategies for the production of isotopically labelled Fab fragments of therapeutic antibodies in Komagataella phaffii (Pichia pastoris) and Escherichia coli for NMR studies
Source: PLoS One. 2023 Nov 29;18(11):e0294406. doi: 10.1371/journal.pone.0294406 (PMC10686436; doi:10.1371/journal.pone.0294406)
Supplement: S2 Raw images — (PDF) [file pone.0294406.s010.pdf]

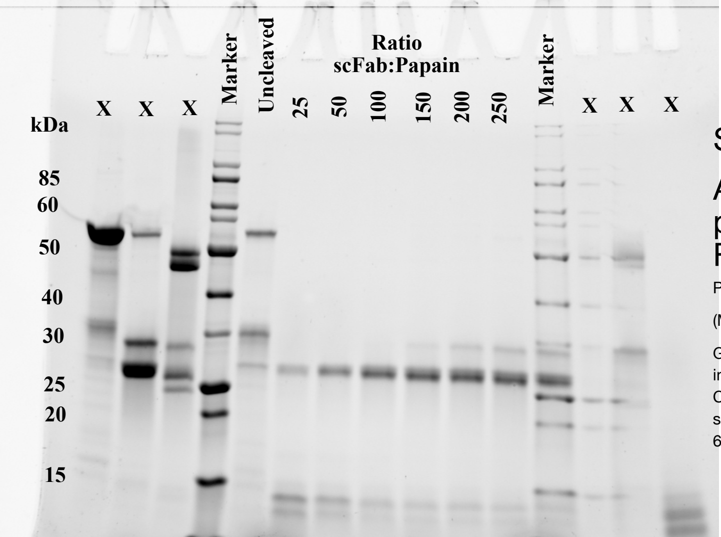

S4_fig panel A

Adalimumab papain cleavage Ratios

Precast stain-free gels

(Mini-PROTEIN TGX, any kD)

Gels were activated and imaged by BIO-RAD ChemiDoc MP imaging system using the Image Lab 6.1 software.

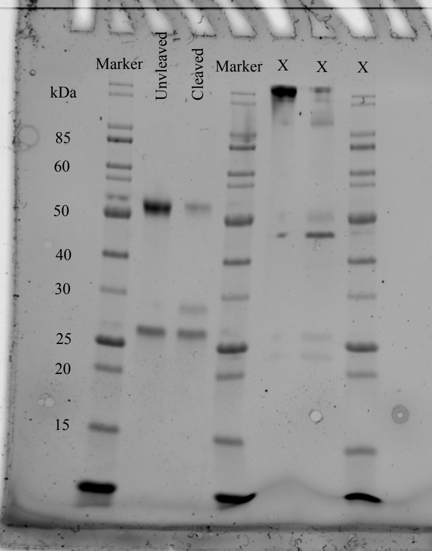

S4_fig panel B

Rituxan papain cleavage 16h

Precast stain-free gels

(Mini-PROTEIN TGX, any kD)

Gels were activated and imaged by BIO-RAD ChemiDoc MP imaging system using the Image Lab 6.1 software.

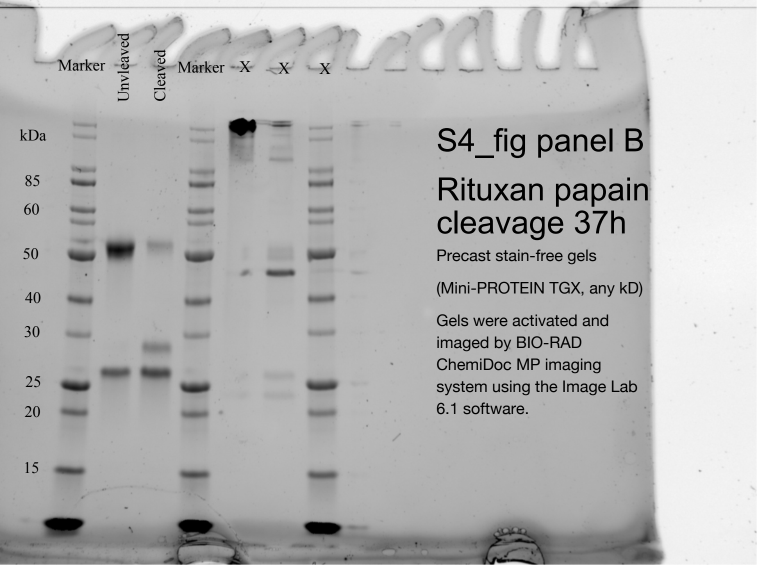
Marker
Unvleaved
Cleaved
Marker
X
X
X
kDa
85
60
50
40
30
25
20
15
S4_fig panel B
Rituxan papain cleavage 37h
Precast stain-free gels
(Mini-PROTEIN TGX, any kD)
Gels were activated and imaged by BIO-RAD ChemiDoc MP imaging system using the Image Lab 6.1 software.

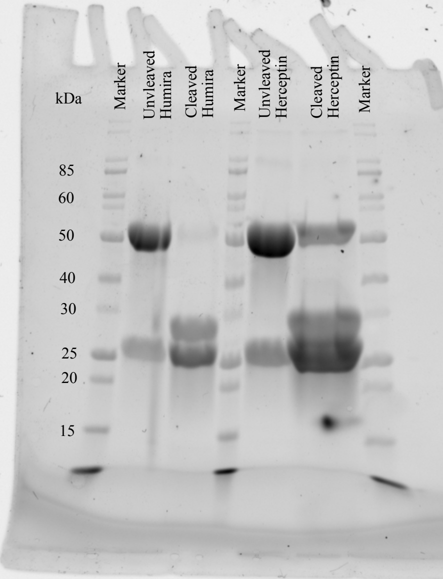

S4_fig panel B

Humira and Herceptin papain cleavage

Precast stain-free gels

(Mini-PROTEIN TGX, any kD)

Gels were activated and imaged by BIO-RAD ChemiDoc MP imaging system using the Image Lab 6.1 software.
